# Collagen epitope expression on B cells is sufficient to confer tolerance to collagen-induced arthritis

**DOI:** 10.1186/s13075-016-1037-7

**Published:** 2016-06-14

**Authors:** Sofia E. M. Andersson, Tove Eneljung, Sara Tengvall, Pernilla Jirholt, Anna Stern, Louise Henningsson, Bibo Liang, Katrin Thorarinsdottir, Jan Kihlberg, Rikard Holmdahl, Inga-Lill Mårtensson, Kenth Gustafsson, Inger Gjertsson

**Affiliations:** Department of Rheumatology and Inflammation Research, Institute for Medicine, Sahlgrenska Academy, University of Gothenburg, Box 480, SE 405 30 Gothenburg, Sweden; Medical Inflammation Research, Department of Medical Biochemistry and Biophysics, Karolinska Institutet, Solna, Sweden; Southern Medical University, Guangzhou, People’s Republic China; Department of Chemistry, BMC, Uppsala University, Uppsala, Sweden; Molecular Immunology Unit, UCL Institute of Child Health, London, UK; Sahlgrenska University Hospital, Gothenburg, Sweden

**Keywords:** Tolerance, B cells, B lymphocytes, Arthritis, Collagen, Collagen type II, Gene therapy, Antigen presentation

## Abstract

**Background:**

The mechanisms underlying tolerance induction and maintenance in autoimmune arthritis remain elusive. In a mouse model of rheumatoid arthritis, collagen type II (CII)-induced arthritis, we explore the contribution of B cells to antigen-specific tolerance.

**Methods:**

To generate expression of the CII-peptide specifically on B-cell major histocompatibility complex type II, lentiviral-based gene therapy including a B-cell-specific Igk promoter was used.

**Results:**

Presentation of the CII-peptide on B cells significantly reduced the frequency and severity of arthritis as well as the serum levels of CII -specific IgG antibodies. Further, both frequency and suppressive function of regulatory T cells were increased in tolerized mice. Adoptive transfer of regulatory T cells from tolerized mice to naïve mice ameliorated the development of CII-induced arthritis.

**Conclusion:**

Our data suggest that endogenous presentation of the CII-peptide on B cells is one of the key contributors to arthritis tolerance induction and maintenance.

**Electronic supplementary material:**

The online version of this article (doi:10.1186/s13075-016-1037-7) contains supplementary material, which is available to authorized users.

## Background

The pathogenesis of rheumatoid arthritis (RA) is complex and not fully understood. A hallmark of RA is the production of autoantibodies to citrullinated proteins and to IgG (rheumatoid factor). These are present in serum and produced in the joints [1–3]. Antibodies are produced by B cells, and during the last decade this cell type has regained interest as a pathogenic cell because autoantibody production precedes the onset of RA [4]. Moreover, therapies that target B cells by either depletion or decreased survival (rituximab, anti-BAFF) have been introduced as successful treatment strategies [5]. B cells do not only play a role as (auto)antibody-producing cells, however, because in experimental models these cells have also been suggested to have a regulatory function, associated with their production of, for example, IL-10 and IL-35 [6–8]. Further, B cells have the capacity to induce regulatory T cells (Tregs) [9] and B-cell specific genes are up-regulated in transplantation tolerance [10]. Harnessing the regulatory role of B cells could thus potentially be used to re-establish tolerance.

The most commonly used mouse model of RA is collagen-induced arthritis (CIA). This model is dependent on the presentation of a defined collagen type II (CII) peptide (amino acids 259–270) on the major histocompatibility complex type II (MHC II) allele A^q^ on antigen-presenting cells (APCs) and subsequent activation of antigen-specific T cells and B cells [11]. The B-cell response is crucial for development of CIA, as B-cell-deficient mice are resistant to CIA [12] and B-cell depletion before immunization delays development of CIA [13].

Oral feeding with CII prior to induction of CIA has been shown to ameliorate arthritis development [14], but it may also enhance the disease [15]. A more pronounced and specific suppression of arthritis and hence CII immune tolerance has been achieved by intravenous injection of a fusion protein consisting of MHC II and glycosylated CII-peptide amino acids 259–270 [16]. However, it is still not known whether B cells are suitable for tolerance induction in CIA. This study aims to investigate whether expression of the CII-peptide, loaded onto A^q^ on B cells, induces CII tolerance and regulates CIA.

We have shown previously that administration of lentiviral particles which results in expression of the CII-peptide on all APCs induces tolerance [17–19]. Here, we use our lentiviral system to target expression of the CII-peptide specifically on B cells, using a B-cell specific Igk promoter [20]. We show that endogenous presentation of the CII-peptide on B cells initiates and sustains tolerance to CIA. The results suggest that antigen presentation by B cells is sufficient to confer tolerance in CIA, and the model provides new insights into tolerance mechanisms.

## Methods

### Lentiviral vectors

**Fig. 1:**
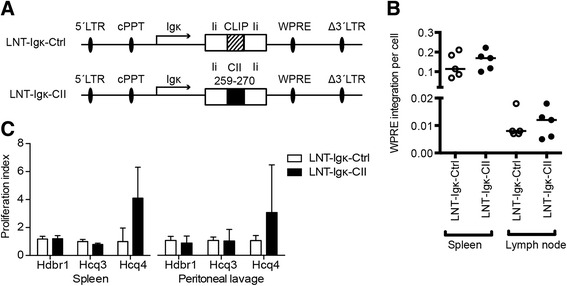
Lentiviral vector design, integration and expression of the CII-peptide on MHC II*.* **a** Lentiviral vectors with an Igk promoter and the collagen type II (*CII*) amino acids 259–270 peptide cloned into the Ii (*LNT-Igk-CII*) and the control vector with the original class II-associated invariant chain peptide (*CLIP*) sequence (*LNT-Igk-Ctrl*). *LTR* long terminal repeat, *WPRE* woodchuck post-transcriptional regulatory element, *cPPT* central polypurine tract. **b** Confirmation of vector integration, detected as WPRE DNA fragment, in cells from spleen and lymph from recipient mice 22 weeks after intravenous injection of transduced CD34^+^ cells. **c** Proliferation index of 5 × 10^5^ T-cell hybridomas specific for hydroxylated (Hdbr1), glycosylated (Hcq3) and naked (Hcq4) CII-peptide co-cultured with 5 × 10^6^ Igk-CII cells from spleen and peritoneal lavage

Generation of the CII-expressing lentiviral vector (LNT-SFFV-CII/Ctrl; Additional file 1: Figure S1) has been described previously [17]. Briefly, CII amino acids 259–270 was cloned into the class II-associated invariant chain peptide position (CLIP) position of the invariant chain (Ii) to achieve efficient loading of and binding to MHC II. As a control vector, the original CLIP sequence was kept in Ii. To generate a specific expression of the CII-peptide on B-cell A^q^, the Ii fragments were subcloned into the lentiviral vector pHR′SIN-cPPT-SFFV with an Igk promoter for B-cell specific expression [20] and were named LNT-Igk-CII and LNT-Igk-Ctrl (Fig. 1a). The linker AGCTTCAATTGTACGTACTCGAGCCGC was added to plasmid Igk-E-eGFP between the *Hind*III and *Sac*II sites, cleaved with *Mfe*I and *Xho*I, and ligated to the Ii-CII and Ii-CLIP fragments isolated from pIi-CII and pIi-CLIP following digestion with *Eco*RI and *Xho*I. The lentiviral vector pTZ18R was ligated to linker AGCTCAATTGGTACCTACGTACTCGAGACTAGT between *Hind*III and *Eco*RI. Subsequently, the Igk promoter and the plasmids containing Ii-CII and Ii-CLIP were digested with *Kpn*I and *Xho*I and were ligated into the *Kpn*I and *Xho*I sites in the linker in pTZ18R, resulting in LNT-Igk-CII and LNT-Igk-Ctrl.

Sequencing was performed on the Ion Torrent platform (Thermo Fisher Scientific, Carlsbad, CA, USA) to confirm the plasmid sequence. Purified plasmid (1 μg) was sheared and size selected to 200 base pairs (bp) using the Ion Xpress Plus Fragment Library Kit in a Library Builder instrument (Thermo Fisher Scientific). A suitable dilution of the template was calculated after quantification using the Ion Library quantitation kit (Thermo Fisher Scientific). The diluted library was loaded on an Ion One Touch 2 instrument (Thermo Fisher Scientific) using the 200 bp chemistry kit to perform emulation PCR on Ion Sphere particles, which were loaded on an Ion 314 chip v2. Sequencing was then performed with the Hi-Q Sequencing Kit on an Ion personal genome machine (PGM; Thermo Fisher Scientific) using default parameters in Ion Torrent Suite version 4.6. The obtained fastaq sequence files were imported into the CLC Genomics Workbench software (QIAGEN Aarhus, Denmark) to create a consensus sequence after mapping to a reference sequence representing the vector construct as well as by de-novo analysis (Additional file 2: Figure S2): LNT-Igk-CII [GenBank:KU879253] and LNT-Igk-Ctrl [GenBank:KU879254].

### Production of lentiviral particles

Vesicular stomatitis virus G pseudotyped lentivirus was produced by transient transfection of 293T cells with three plasmids—the self-inactivating transfer vector plasmid LNT-Igk-CII, LNT-Igk-Ctrl, LNT-SFFV-CII or LNT-SFFV-Ctrl, the multi-deleted packaging plasmid; pCMVΔR8.74 and the VSV-G envelope; or pMD.G2—and titrated as described previously [18].

### Mice

Male DBA/1 mice, 6–8 weeks old, were obtained from Taconic (Europe A/S, Ry, Denmark) and housed in a pathogen-free barrier facility (12-h light/12-h dark cycle) and fed rodent chow. The local Animal Ethics Committee approved all animal studies (numbers, 105-2009 and 277-2011).

### Transplantation of haematopoietic stem cells

Both donor and recipient mice were treated with Baytril® (0.6 mg/ml) in the drinking water before transplantation, and the treatment continued for the recipients 2 weeks after transplantation. Bone marrow cells were harvested from the femur and os ilium of DBA/1 mice and haematopoietic stem cells (HSCs) were purified using the EasySep™ Mouse Hematopoietic Progenitor Cell Enrichment Kit (Stemcell Technologies, Manchester, UK). Purified HSCs were cultured overnight under standard conditions in StemSpan expansion medium (Stemcell Technologies) with 100 ng/ml mSCF, 100 ng/ml mFlt3L, 100 ng/ml IL-11, 20 ng/ml IL-3 (R&D Systems, Abingdon, UK) and lentiviral particles at multiplicity of infection 75 (LNT-SFFV-CII/Ctrl) or 40 (LNT-Igk-CII/Ctrl). The following day, cells were re-suspended and washed before intravenous injection of 2.5 × 10^5^ cells into syngeneic lethally irradiated (8.5 Gray) recipient naïve mice. The cells were allowed to repopulate the mice for a minimum of 10 weeks before induction of CIA or adoptive transfer into naïve syngeneic recipient mice. The arthritis experiments using the Igk promoter system were repeated independently three times with a total of *n* = 26 LNT-Igk-Ctrl mice and *n* = 25 LNT Igk-CII mice and terminated at various time points. Adoptive transfer of Tregs from LNT-Igk-CII/Ctrl mice and B cells from mice transplanted with LNT-SFFV-CII/Ctrl was performed once.

### Confirmation of vector integration and Ii-CII expression

To confirm the presence of vector integration in spleen and lymph node cells 22 weeks after bone marrow transplantation, DNA was extracted from lymph nodes using the Allprep DNA/RNA mini prep kit and from splenocytes using the QIAamp DNA minikit (Qiagen). WPRE sequences were amplified by Taqman Universal PCR (Applied Biosystems, Carlsbad, CA, USA) and normalized to the amplification of the titin gene, as described previously [21].

### Detection of CII protein expression on MHC II

To investigate presentation of the CII-peptide on MHC II molecules, T-cell hybridomas specific for modified CII-peptides were used: Hcq3 hybridoma (recognizes glycosylated CII amino acids 259–270), Hcq4 (recognizes naked CII amino acids 259–270) and HdBr1 (recognizes hydroxylated CII amino acids 259–270). To ensure proper specificity of the hybridoma, 5 × 10^4^ cells from each hybridoma were co-cultured in a 96-well plate for 24 hours with 5 × 10^5^ splenocytes that were primed with 20, 10, 5, 2.5 μg/ml naked CII-peptide, 20, 5, 1.25, 0.4 μg/ml hydroxylated CII-peptide or 20, 5, 1.25, 0.4 μg/ml glycosylated CII-peptide. Analysis of IL-2 levels in supernatants was performed using ELISA (R&D Systems) and showed specific peptide responses for each hybridoma (Additional file 1: Figure S1). The specific T-cell hybridomas were then used to investigate CII-peptide expression on A^q^ on cells from the peritoneal cavity and spleen 12 weeks after transplantation. Peritoneal cavities were flushed with PBS to harvest peritoneal cells and the spleen was removed from transplanted mice (*n* = 3 per group). The spleens were squeezed through a 70 μm cell strainer. After spinning and washing, the cells were counted (Nucleocounter; ChemoMetec A/S, Allerød, Denmark) and seeded at 2.5 × 10^6^ cells/ml on a 96-well plate in duplicate and co-cultured with T-cell hybridomas at 2.5 × 10^5^ cells/ml for 24 h. The levels of IL-2 in culture supernatants were measured by ELISA.

### Collagen-induced arthritis

The mice were immunized in the tail base with rat CII (1 mg/ml) and complete Freund’s adjuvant (CFA; Sigma-Aldrich, Stockholm, Sweden) in a total volume of 100 μl and boosted 21 or 28 days later with CII (1 mg/ml, 100 μg/mouse) in incomplete Freund’s adjuvant (Sigma-Aldrich). The intervals between the primary immunization and booster vary due to a change in the ethical permit. Arthritis severity and frequency were graded blindly at the indicated time points after CII immunization. All of the mice were followed up individually and arthritis (defined as visible erythema and/or joint swelling) was evaluated by inspection of finger/toe and ankle/wrist joints. Severity of arthritis was evaluated by macroscopic inspection of each limb, and a score was assigned for each limb: 0, neither swelling nor erythema; 1, mild swelling and/or erythema; 2, moderate swelling and erythema; and 3, marked swelling and erythema. The total score for each mouse was calculated by adding up the scores for each limb.

### Histological examination of inflamed joints

Histopathologic examination of the joints was performed after routine fixation, decalcification and paraffin embedding. Tissue sections from fore and hind paws were cut and stained with haematoxylin and eosin. All the slides were coded and evaluated by two blinded observers. The specimens were evaluated with regard to synovial hypertrophy, leukocyte infiltration and cartilage/bone destruction. The degree of synovitis and destruction in every joint region (finger/toes, wrists/ankles, elbows and knees) was assigned a score from 0 to 3. Occasionally, one paw was missing in the histological sections, or embedded in such a way that it was impossible to evaluate the degree of synovitis and bone/cartilage destruction. Therefore, the total score per mouse was divided by the number of joints evaluated.

### Flow cytometry

Single cell suspensions were prepared from the spleen and blood. Spleens were filtrated through cell strainers before washing and centrifugation for 10 min at 1500 rpm. The blood was collected in Eppendorf tubes containing heparin. Erythrocytes were lysed using lysis buffer (0.16 M NH_4_Cl, 0.13 mM EDTA, 12 mM NaHCO_3_). Cells were resuspended in PBS with 10 % FCS, counted in a Nucleocounter and seeded in 96-well plates. Fcγ receptors were blocked (clone 2.4G2; BD Biosciences, Franklin Lakes, New Jersey, USA) before application of surface antibodies. T cells were stained using CD4 (V450 clone RM4-5; BD Biosciences) and CD25 (FITC, clone 3C7; BD Biosciences) for 20 min at room temperature (RT). Intracellular staining was performed using FoxP3/Transcription Factor Staining Buffer set (eBioscience, San Diego, USA) and FoxP3 (phycoerythrin (PE), clone NRRF-30; eBioscience, San Diego, USA) or an isotype control (PE, clone eBR2a) for 30 min at 8 °C. Cells were detected by FACSCanto II™ (BD Biosciences) and analysis was performed by FlowJo Software (Tree Star Inc., Ashland, OR, USA). Gates for both surface and intracellular staining were set according to flourochrome minus one settings [22].

### CII-specific IgG and IgM antibodies and total IgG antibodies in serum

Levels of CII-specific IgG in serum, collected at indicated time points after CII immunization, were determined by ELISA. Low binding plates (NUNC, Fisher Scientific, Gothenburg, Sweden) were coated with rat CII (1 μg/ml) in PBS. Serum samples were diluted (1/7500, 1/22,500, 1/67,500 and 1/202,500) and after incubation CII-specific IgG was detected by biotinylated rat anti-mouse IgG, IgG1, IgG2a or IgG2b at 0.5 μg/ml (Serotec, Oxford, UK) or biotinylated (Fab)_2_ goat anti-mouse IgM (Jackson ImmunoResearch Laboratories, West Grove, PA, USA). The assays were developed using extravidin–horseradish peroxidase (HRP) and tetramethylbenzidine substrate. The reactions were stopped with H_2_SO_4_ and read in Spectra Max 340PC (Molecular Devices, Sunnyvale, CA, USA) at 450 nm with correction at 650 nm.

### Anti-recombinant CII epitope ELISA assay

A 96-well plate was coated with anti-recombinant CII-peptides at 5 μg/ml, 100 μl/well and incubated at 4 °C overnight. The plate was blocked with 0.3 % milk powder for 2 h at RT. Serum diluted 1:100 was added to the plate and incubated for 2 h at RT. Rat Anti-Mouse Kappa-HRP (clone 187.1; Southern Biotech, Bromma, Sweden) was added at dilution 1:4000. The assay was developed using ABTS substrate and buffer (Roche Life Science, Bromma, Sweden) and read at 405 nm.

### *Mycobacterium tuberculosis* IgG ELISA

Heat-killed *Mycobacterium tuberculosis* H37Ra (Difco, BD Biosciences, Franklin Lakes, New Jersey, USA) 0.4 mg/ml was dissolved in carbonate buffer, and filtered through a 22 μm Millipore filter. A 96-well plate (Nunc Maxisorp) was coated with 100 μl per well of the *M. tuberculosis* solution and incubated at 4 °C overnight and further blocked with PBS with BSA 1 %, Tween 1 %. The serum was serially diluted from 1:8 to 1:512 and the plate was incubated at 4 °C overnight. Biotinylated goat anti-mouse IgG (Jackson) was added at dilution 1:3000. The assays were developed using streptavidin–HRP (R&D) and tetramethylbenzidine substrate. The plate was read at 450 nm.

### Immunofluorescent staining of tissue sections

Mouse spleen tissue embedded in OCT (Histolab, Västra Frölunda, Sweden) was snap-frozen using dry ice. Frozen tissue was cut in 7 μm thick sections on a Leica CM3050 cryostat. Tissue slides were fixated in ice-cold ethanol for 10 min and allowed to air dry, then either stained immediately or kept at –20 °C until staining. Slides were put in PBS for 5 min to remove OCT and then mounted into a Shandon cover plate Sequenza slide rack (Thermo Fisher Scientific, Västra Frölunda, Sweden). Unspecific binding was blocked using diluted horse serum or DAKO protein block. Antibodies for detection of surface markers rat anti-mouse B220 (biotinylated, RA3-6B2; BD Pharmingen, Franklin Lakes, New Jersey, USA), hamster anti-mouse TCRβ (Alexa Fluor 488, H57-597; Biolegend, San Diego, CA, USA) and rat anti-mouse GL7 (e660 GL-7; eBioscience, San Diego, USA) were diluted in PBS and applied to sections, and incubated overnight at 4 °C. For intracellular staining of Foxp3, sections were incubated with 4 % PFA in PBS for 5 min, then 0.5 % Triton-X-100 for 5 min, blocked with protein block (DAKO, Stockholm, Sweden) and then stained with rat anti-mouse foxp3 (e570, FKJ-16 s; eBioscience, San Diego, USA) diluted in 0.1 % saponin in PBS. Slides were washed three times with PBS–saponin and mounted with fluorescent mounting medium (DAKO, Stockholm, Sweden). Images were acquired on a Zeiss LSM 700 confocal image fluorescent microscope with ZEN 2009 acquisition software (Zeiss, Oberkochen, Germany) at 1.6 pixels per μm. The number of GL7 clusters were counted and divided by the number of follicles per slide. A mean of duplicate slides was calculated per individual mouse.

### T-cell suppression assay

Single cell suspensions were prepared from the spleen and lymph nodes of LNT-Igk-CII/Ctrl mice (*n* = 3 per group). Erythrocytes were depleted from single cell suspensions using anti-TER 119 micro beads (Miltenyi Biotech). Cell suspension was then stained for surface expression of anti-CD4 (allophycocyanin (APC), RM4-5; BD Biosciences) and anti-CD25 (PE, PC61; BD Biosciences) and sorted into a CD4^+^CD25^+^ Treg population using a FACS Sy3200 Sorter (Sony Biotechnology, Champaign, IL, USA). Tregs were expanded for 8 days using a Treg Expansion Kit (Miltenyi Biotech Norden AB, Lund, Sweden). MHC II^+^ APCs and CD4^+^CD25^–^ effector T cells were sorted from the spleen of naïve DBA/1 mice. Before sorting, erythrocytes were depleted from single cell suspensions using anti-TER 119 micro beads (Miltenyi Biotech) and stained with anti-CD4 (APC, RM4-5; BD Biosciences), anti-CD25 (PE, PC61; BD Biosciences) and anti-MHC II (APC-e780; eBioscience, San Diego, USA). Sorted Tregs were stained with 10 μM CellTrace violet at 37 °C. APCs (5 × 10^4^), effector T cells (2 × 10^4^) and Tregs (according to ratio) were seeded in DMEM (supplemented with 10 % FCS, 1 % 2-mercaptoethanol, 1 % penicillin/streptomycin) with added anti-CD3 (azide free; BD Biosciences) at a final concentration of 0.5 μg/ml in a 96-well plate. After 6 days, proliferation was evaluated by CellTrace violet fluorescence of cells acquired on a FACSCanto II™.

### Adoptive transfers

Adoptive transfer of CD4^+^CD25^+^ T cells from LNT-Igk-Ctrl/CII mice to naïve recipients was carried out before CII immunization. Eighteen weeks after transplantation, CD4^+^CD25^+^ cells were sorted from spleen and lymph node cells of LNT-Igk-CII/Ctrl mice (*n* = 4 per group) using a FACS Sy3200 Sorter (Sony Biotechnology). Erythrocytes were depleted from single cell suspensions using anti-TER 119 micro beads (Miltenyi Biotech) and the remaining cells were stained with anti-CD4 (APC, RM4-5; BD Biosciences) and anti-CD25 (PE, PC61; BD Biosciences). Following 30 min of incubation at 4 °C, cells were washed and sorted by FACS (Additional file 3: Figure S3A). Purity and viability of isolated cells was around 90–95 % (Additional file 3: Figure S3B). Tregs were expanded for 8 days using a Treg Expansion Kit (Miltenyi Biotech). CD4^+^CD25^+^ T cells (5.5 × 10^5^ per mouse) were adoptively transferred into naïve syngeneic recipient mice 2 days before CII immunization and arthritis development was evaluated as already described.

CD19^+^MHC II^+^ B cells from LNT-SFFV-CII/Ctrl mice were transferred to naïve DBA/1 mice 28 days after CII immunization. Fourteen weeks after transplantation of LNT-SFFV-CII/Ctrl transduced HSCs, B cells (defined as both CD19^+^ and MHC II^+^ cells) were sorted by FACS Synergy (Additional file 3: Figure S3C). Briefly, single cell suspensions were prepared in PBS with 10 % FCS. APC-Cy7 Rat Anti-Mouse CD19 (BD Pharmingen) and eFlour450 Anti-mouse MHC class II (I-A/I-E) (eBioscience, San Diego, USA) were added to the cell suspensions and, following a 30-min incubation at 4 °C, cells were washed and then sorted by FACS. The freshly isolated B cells (2 × 10^6^) were adoptively transferred into syngeneic recipient mice 28 days after CII immunization and evaluated for development of arthritis as already described.

### Statistical analysis

Statistical analyses were performed using GraphPad Prism (La Jolla, CA, USA). Statistical differences between parametrically distributed groups were calculated using Student’s *t* test, with a Bonferroni correction for multiple comparisons when applicable, and for two-variable datasets a two-way ANOVA was used. Differences between non-parametrically distributed groups were calculated using the Mann–Whitney U test for quantitative data and Fisher’s exact test for nominal data. Linear regression was used to compare development of severity of arthritis between treatment groups. *P* < 0.05 was considered significant.

## Results

### B cells express the CII-peptide in its naked non-modified form

Presentation of the immunodominant T-cell epitope in CII, the CII-peptide amino acids 259–270, on MHC II A^q^ is important for both disease development [23, 24] and tolerance [16, 17, 25]. We have shown previously that adoptive transfer of B cells presenting the CII - peptide on MHC II A^q^ induces partial tolerance to CIA [19]. In these experiments we used the LNT-SFFV-CII/CLIP lentiviral vectors, where expression of CII was driven by the general SFFV promoter (Additional file 3: Figure S3B). To test the hypothesis that B cells are important mediators of antigen-specific tolerance, we generated a lentiviral vector where the expression of the CII-peptide was driven by the B-cell specific k light chain (Igk) promoter [20] (LNT-Igk-CII) and a control vector containing the native CLIP peptide (LNT-Igk-Ctrl) (Fig. 1a). The expected efficacy of the Igk promoter is transgene expression in around 10 % of B cells [20]. To express the CII-peptide on MHC II A^q^, we transduced HSCs with the selected lentiviral particles and subsequently injected the HSCs into lethally irradiated recipient mice. The mice were allowed to reconstitute their haematopoietic cell populations for at least 10 weeks before cells were harvested or CIA was induced.

Vector integration was confirmed by quantitative PCR analysis of the woodchuck post-transcriptional regulatory element (WPRE) DNA sequence in cells from the spleen and lymph node from LNT-Igk-CII and LNT-Igk-Ctrl after HSC reconstitution (Fig. 1b). Protein expression of the CII-peptide on B-cell A^q^ was verified using CII-specific T-cell hybridomas recognizing post-translational modified forms of the CII-peptide [26]. We found that all hybridomas were functional and responded to the expected peptide (Additional file 1: Figure S1A) and that the CII-peptide, predominantly the naked non-modified variant, was weakly expressed on cells from both spleen and peritoneal lavage (Fig. 1c).

### Frequency and severity of arthritis is reduced in LNT-Igk-CII mice

**Fig. 2:**
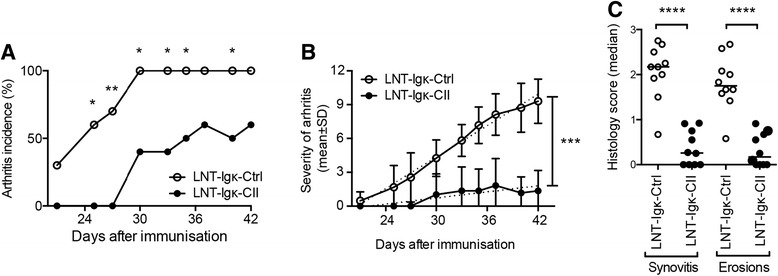
CIA development in LNT-Igk-Ctrl and LNT-Igk-CII mice. **a** Arthritis frequency and **b** clinical severity after CIA induction in LNT-Igk-Ctrl and LNT-Igk-CII mice. **c** Histopathological evaluation of synovitis and joint erosions at day 40 after CIA induction (*n* = 10 per group, one of three experiments is shown). Indicated *P* values were determined using Fisher’s exact test for nominal data, linear regression to compare the severity of arthritis (shown as mean ± SD) and a non-parametric test for categorical data. *P < 0.05; **P < 0.01; ***P < 0.001

Clinical evaluation of arthritis showed a delayed onset and reduced frequency of arthritis in LNT-Igk-CII mice (53 %) compared with LNT-Igk-Ctrl mice (95 %) (Fig. 2a). The severity of arthritis was also greatly reduced in LNT-Igk-CII mice compared with LNT-Igk-Ctrl mice and remained at this low level throughout the course of arthritis (Fig. 2b). The histological scores showed a considerably milder pathology in LNT-Igk-CII mice with reduced synovitis and erosion score compared with LNT-Igk-Ctrl mice (Fig. 2c), hence confirming the clinical evaluation of arthritis. However, when CII-expressing B cells from non-immunized transplanted mice were transferred to mice after CII immunization, no effect on arthritis development was detected (Additional file 1: Figure S1C).

### LNT-Igk-CII mice display reduced levels and a limited repertoire of CII-specific IgG antibodies

**Fig. 3:**
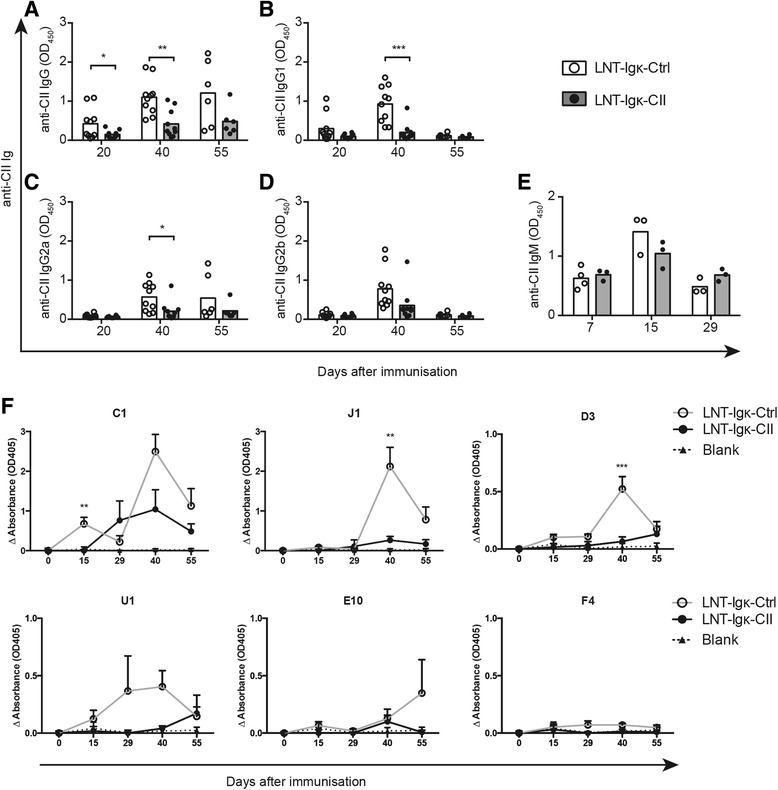
Anti-CII IgG and IgM in serum from LNT-Igk-Ctrl and LNT-Igk-CII mice. Serum levels of **a** anti-CII IgG, **b** anti-CII IgG1, **c** anti-CII IgG2a, **d** anti-CII IgG2b and **e** anti-CII IgM in LNT-Igk-Ctrl and LNT-Igk-CII mice measured by ELISA at day 20, day 40 (*n* = 10 per group) and day 55 (*n* = 6 per group) after immunization. **f** Change in serum levels of the IgG antibodies to the specific CII epitopes (C1, J1, D3, U1, E10 and F4) measured as Δabsorbance (absorbance at indicated day – mean value at day 0). Serum was obtained from LNT-Igk-Ctrl and LNT-Igk-CII mice at day 0 (*n* = 3 per group), day 15 (*n* = 6 per group), day 40 (*n* = 10 per group) and day 55 (*n* = 7 and 6, respectively). Data shown as mean ± SEM. Indicated *P* values were determined using a two-tailed *t* test with a Bonferroni correction for multiple comparisons when comparisons were made for multiple anti-recombinant CII-peptide assays. *P < 0.05; **P < 0.01; ***P < 0.001

As development of CIA is dependent on CII-specific antibodies [27, 28], serum levels of CII-specific antibodies were determined at the indicated time points. CII-specific IgG antibodies were detected in all animals after immunization, but the levels were significantly decreased in LNT-Igk-CII mice compared with LNT-Igk-Ctrl mice with the highest level 40 days after immunization (Fig. 3a). At this time point, the levels of CII-specific IgG1 and IgG2a were reduced in LNT-Igk-CII mice (Fig. 3b–d). However, there were no differences in serum levels of CII-specific IgM antibodies between the LNT-Igk-CII or LNT-Igk-Ctrl mice at days 0 or at days 7, 15 and 29 days after immunization (Fig. 3e). CII expression on B cells thus impaired the IgG but not IgM response to CII, indicating that the tolerogenic effect of the B cells involved T cells.

In CIA, the CII-specific IgG response is heterogeneous and results in antibodies to several well-defined CII epitopes. Antibodies directed to epitopes such as C1, J1, D3 and U1 mediate disease [29] while others such as those to F4 are considered protective [30]. We hypothesized that there would be a shift in the antigen specificity between LNT-Igk-Ctrl versus LNT-Igk-CII mice. We determined the delta change in serum levels of CII-specific IgG antibodies targeting these epitopes, from baseline and after immunization. In LNT-Igk-Ctrl mice, IgG antibodies to all CII epitopes except F4 were increased from baseline (day 0). By contrast, the LNT-Igk-CII mice did not mount an IgG response except to the C1 epitope, which was delayed and lower demonstrating that the CII-specific IgG response to disease-mediating epitopes is substantially reduced (Fig. 3f).

### Reduced germinal centre formation in LNT-Igk-CII mice

**Fig. 4:**
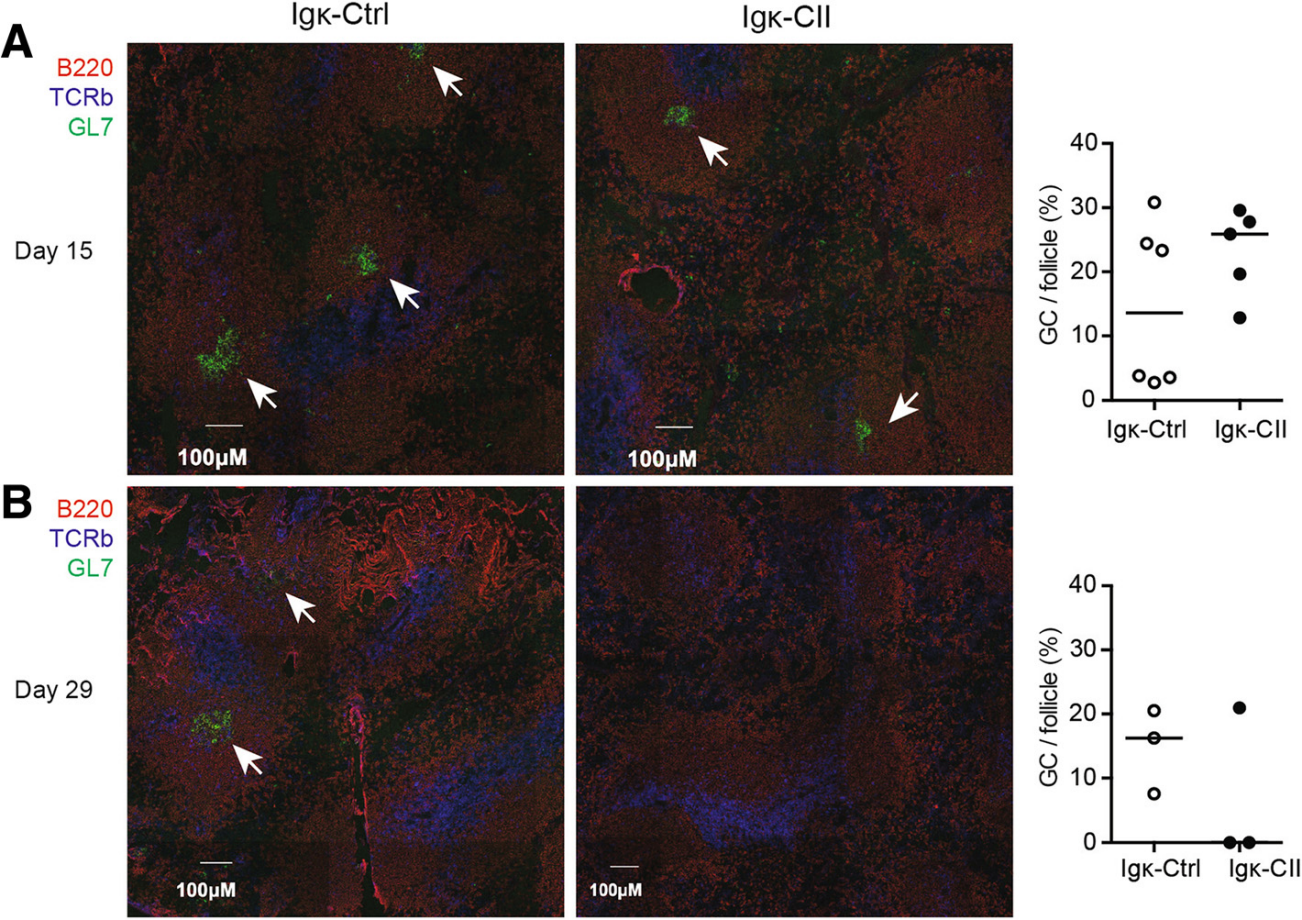
Frequency of GCs in spleens from LNT-Igk-Ctrl and LNT-Igk-CII mice after CIA induction. Frequency of GCs per follicle in spleens **a** 15 days after immunization from LNT-Igk-Ctrl mice (*n* = 6) and LNT-Igk-CII mice (*n* = 6) or **b** 29 days after immunization from LNT-Igk-Ctrl mice (*n* = 3) and LNT-Igk-CII mice (*n* = 3). *GC* germinal centre

The reduction in the CII-specific IgG response in LNT-Igk-CII mice suggested a T-cell-mediated effect, which could result in impaired germinal centre (GC) formation. The frequency of splenic GCs was determined at day 15 after immunization and found to be similar in both LNT-Igk-and LNT-Igk-Ctrl mice (Fig. 4a). As both groups of mice mounted an IgG response to *M. tuberculosis*, which is a part of Freund’s complete adjuvant, the GC formation in spleen after the primary immunization (Additional file 1: Figure S1D) at day 15 does not have to be CII specific. However, at day 29 (i.e. the day after CII booster (in incomplete adjuvant)) only one mouse out of three in the LNT-Igk-CII group displayed GC formation, at the same time as the corresponding numbers in the control group were three out of three mice (Fig. 4b). These results indicate that B-cell specific expression of the CII-peptide in LNT-Igk-CII mice might have an effect on the T-cell-dependent immune response to CII.

### Frequency and suppressive function of Tregs are enhanced in LNT-Igk-CII mice

**Fig. 5:**
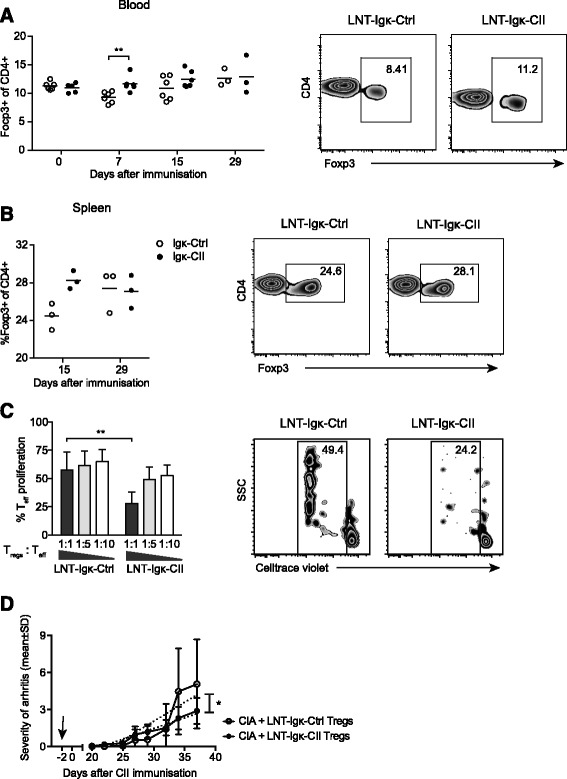
Frequency and suppressive capacity of Tregs. Treg frequency in **a** blood and **b** the spleen from LNT-Igk-Ctrl and LNT-Igk-CII mice at indicated time points during CIA. Indicated *P* values are calculated using Student’s *t* test. **c** Proliferation of Teffs (CD4^+^CD25^–^) from naïve DBA1 mice, stimulated for 6 days with APCs and αCD3 in the presence of Tregs (CD4^+^CD25^+^) from LNT-Igk-CII mice (*n* = 3) and LNT-Igk-Ctrl mice (*n* = 3) in Tregs: Teff ratio 1:1 (flow cytometry plot), 1:5 and 1:10. Two-way ANOVA was used to calculate *P* values. **d** Clinical severity of arthritis in mice that received Tregs from LNT-Igk-Ctrl mice (*n* = 5) or LNT-Igk-CII mice (*n* = 6) 2 days before CIA induction. The indicated *P* value is calculated using a comparison between slopes after linear regression and data are shown as mean ± SD. *P < 0.05; **P < 0.01; ***P < 0.001. *CIA* collagen-induced arthritis, *CII* collagen type II, *Teff* effector T cell, *Treg* T regulatory cell

We have shown previously that tolerance induced by presentation of the CII-peptide on A^q^, driven by a general promoter, is associated with an increased proportion of Tregs [18, 19], which is consistent with Tregs as key mediators of peripheral tolerance and their ability to prevent the development of CIA [31]. We therefore hypothesized that presentation of the CII-peptide on B cells in LNT-Igk-CII mice had an effect on Tregs. Determining the frequency of CD4^+^Foxp3^+^ Tregs in blood showed that it was similar in LNT-Igk-Ctrl and LNT-Igk-CII mice before immunization but increased significantly in LNT-Igk-CII mice after immunization, at day 7 in blood (Fig. 5a) and the same trend was seen at day 15 in spleen (Fig. 5b). Moreover, the CD4^+^CD25^+^ Tregs from LNT-Igk-CII mice were also functionally more potent, as they suppressed proliferation of CD4^+^CD25^–^ effector T cells more than did CD4^+^CD25^+^ Tregs from LNT-Igk-Ctrl mice (Fig. 5c).

### Tolerogenic effect in LNT-Igk-CII mice is partly mediated by T cells

To confirm the antigen specificity of the Tregs in LNT-Igk-CII mice, CD4^+^CD25^+^ was adoptively transferred to naïve DBA/1 recipients 2 days before induction of arthritis. The severity of arthritis was reduced in mice that received CD4^+^CD25^+^ from LNT-Igk-CII mice compared with those that received CD4^+^CD25^+^ Tregs from LNT-Igk-Ctrl mice (Fig. 5d).

## Discussion

In this study we demonstrate that expression of the CII-peptide on B cells in the context of MHC II A^q^ is sufficient to induce tolerance in the CIA model. Our results suggest that the presentation of the CII-peptide on B cells increases the frequency and suppressive capacity of Tregs, and leads to decreased levels of CII-specific IgG antibodies and reduced frequency and severity of arthritis.

Our results are supported by previous work implicating B cells in tolerance induction. Antigen presentation by B cells prevents cognate activation of naive, but not memory, T cells [32] and induces tolerance to soluble antigen [33]. B cells can also induce and maintain tolerance by production of anti-inflammatory cytokines such as IL-10, and B cells genetically or otherwise manipulated to produce IL-10 have immunoregulatory properties in autoimmune diseases [6, 7, 21, 34]. Gene therapy reintroducing Wiskott–Aldrich protein in B cells prevented development of autoreactive B-cell clones in Wiskott–Aldrich syndrome patients [35], highlighting the importance of B cells for tolerance.

Development of CIA is dependent on both T cells [36] and B cells [37]. Further, germline-encoded IgM and IgG CII-specific B cells are not eliminated by central tolerance mechanisms [38, 39]. During CIA T-cell help is needed to enhance the polyclonal expansion, but also the generation, of autoreactive IgG CII-specific B cells [36]. CII-specific antibodies with specificity for different CII epitopes seem to have different pathogenicity; anti-F4 IgG antibodies have been suggested to protect against arthritis, while C1, J1, D3, U1 and E10 IgG antibodies mediate arthritis [30]. We show that after CII immunization, LNT-Igk-Ctrl mice but not LNT-Igk-CII mice developed CII-specific IgG antibodies reactive with all measured CII epitopes. However, there was no increase in anti-F4 IgG in LNT-Igk-CII mice after immunization, suggesting that the tolerance observed is not mediated by production of protective antibodies. Rather, the pathogenic CII antibodies that are evident in the control mice are lacking in the LNT-Igk-CII mice.

The initial immunization using complete Freund’s adjuvant contains a number of soluble antigens besides CII that trigger GC formation which leads to *M. tuberculosis* specific IgG antibodies [40]. However, booster exposure to the CII antigen reactivates the CII-specific memory T cells and B cells and leads to rapid GC formation and increased antibody diversity [41] seen in the control, but not in the LNT-Igk-CII mice.

It has become clear that professional APCs, such as dendritic cells but also B cells, are important for development of the Foxp3^+^ Treg compartment by different mechanisms including IL-10, IL-35 and TGF-β production [42]. Intravenous administration of antigen without adjuvant often leads to tolerance, where the B cells passively take up antigen and present on MHC II [43], which in turn appears to promote Treg differentiation [44]. In addition, passive uptake of CII may lead to a weaker T-cell activation [45] than antigen-specific internalization. Regardless of whether our CII-presenting B cells acquire a regulatory phenotype [46] or whether they represent a naïve phenotype when presenting the peptide [47], the results suggest that they promote Treg development and/or T-cell inactivation rather than T-cell activation [9, 44]. Furthermore, adoptive transfer of Tregs from LNT-Igk-CII mice was superior in transferring tolerance, compared with Tregs from LNT-Igk-Ctrl mice. We could not detect an increased frequency of an IL-10-producing regulatory B-cell phenotype or transitional type 2 marginal zone precursor cells (data not shown) in LNT-Igk-CII mice, which would indicate an expansion of Bregs. This was also supported by the fact that adoptive transfer of CII-presenting B cells could not transfer tolerance to already immunized mice.

Both T-follicular helper cells and Tregs are specific for the immunizing antigen, but self-antigen preferentially induces T-follicular regulatory cells over T-follicular helper cells. T-follicular regulatory cells can derive from peripheral Tregs [48]. In our experiments, the frequency of Tregs was not increased before but well after CII immunization, which was followed by decreased production of CII-specific IgG antibodies and suppression of arthritis in the Igk-CII mice. This observation could indicate that the balance between T-follicular helper cells and T-follicular regulatory cells are skewed towards the latter. This might be of importance for the reduced CII-specific IgG response observed in the LNT-IgK-CII mice.

## Conclusions

Taken together, these experiments suggest that the tolerogenic effect of B cells in this model is mediated by T-cell tolerance. The LNT-Igk-CII B cells will most likely induce tolerance in heteroreactive T cells (i.e. T cells recognizing the rat CII-peptide), which will prevent the T-cell response to CII immunization and hence the cross-reactivity leading to an autoreactive response. Antigen presentation of the CII-peptide on B cells thus induces and maintains tolerance, which in part is mediated by Tregs.

## Abbreviations

APC, allophycocyanin; APCs, antigen-presenting cells; bp, base pairs; CD, cluster of differentiation; CFA, complete Freund’s adjuvant; CIA, collagen-induced arthritis; CII, collagen type II; CLIP, class II-associated invariant chain peptide; cPPT, central polypurine tract; Ctrl, control; ELISA, enzyme-linked immunosorbent assay; GC, germinal centre; HRP, horseradish peroxidase; HSC, haematopoietic stem cell; IFA, incomplete Freund’s adjuvant; Ii, Invariant chain; IL, interleukin; LNT, lentivirus; MHC II, major histocompatibility complex type II; PE, phycoerythrin; RA, rheumatoid arthritis; SFFV, spleen focus forming virus; TGF, transforming growth factor; Treg, regulatory T cell; VSV-G, vesicular stomatitis virus G; WPRE, woodchuck post transcriptional element

## Additional files

Additional file 1: is Figure S1 showing (**A**) verification of the functionality of hybridomas shown as IL-2 production by T-cell hybridomas with TCR specificity for hydroxylated (Hdbr1), glycosylated (Hcq3) or naked (Hcq4) modifications of the lysine residue in the CII259-270 sequence. The different hybridomas were co-cultured with splenocytes primed with naked, hydroxylated or glycosylated CII - peptide at varying concentrations. (**B**) Lentiviral vectors with an SFFV promoter and the CII amino acids 259–270 peptide cloned into Ii (LNT-SFFV-CII) or the control vector with the original CLIP sequence (LNT-SFFV-Ctrl). *LTR* long terminal repeat. (**C**) To determine whether B cells expressing CII-peptide could mediate tolerance during ongoing arthritis, sorted B cells were adoptively transferred to recipient mice at day 28 after CII-immunization (mice were boosted at day 21 in this experiment). As expression from the Igk promoter is low [19] B cells from LNT-SFFV-CII (*n* = 7) or LNT-SFFV-Ctrl mice (*n* = 7) were used to generate a higher frequency of CII-expressing B cells. However, in the effector phase of arthritis, the sorted B cells could not hamper development of arthritis. (**D**) *M. tuberculosis* IgG antibodies correlate to the frequency of GCs at day 15, after the primary immunization. (EPS 639 kb) Additional file 2: is Figure S2 showing lentiviral vector maps. (EPS 2084 kb) Additional file 3: is Figure S3 showing FACS plots for adoptive transfer of Tregs or B cells during, and after, sort. (EPS 2857 kb)
